# Spontaneous subarachnoid hemorrhage caused by ruptured aneurysm of basilar trunk perforator: a case report and literature review

**DOI:** 10.1186/s41016-022-00281-5

**Published:** 2022-06-10

**Authors:** Yao Wu, Zhaoliang Li, Dehong Yang, Tao Wu, Ailin Chen, Chungang Dai, Qing Zhu

**Affiliations:** Second Affiliated Hospital of Soochow University, Sanxiang Road 1055, Suzhou, 215004 China

**Keywords:** Basilar trunk, Intracranial aneurysm, Perforator

## Abstract

**Background:**

Aneurysm of basilar perforator was rarely reported in the literature. It is difficult to treat due to its small size and deep-seated location. Excessive treatment may cause complications that resulted from ischemic events of parent perforators. Therefore, it is important to make clinical strategy for such patients to improve the prognosis.

**Case presentation:**

One case, who presented as spontaneous subarachnoid hemorrhage, despite the negative result in computed tomography angiography firstly, was diagnosed angiographically as a ruptured aneurysm of the basilar perforator. A good clinical outcome of the case was achieved during the follow-up after conservative observation for 2 months, as well as the disappearance of previous lesion from angiography.

**Conclusions:**

Aneurysm located at perforator of basilar trunk was rare and difficult to treat. Conservative observation for certain cases with periodic angiography follow-up was considered in order to prevent the patients from potential iatrogenic effects.

## Background

Aneurysms of the perforators of intracranial arteries are rare, and most of them arise from the lenticulostriate artery of middle cerebral artery (MCA) [1]. Due to the rarity of the perforator of basilar artery, the natural history and the optimal therapeutic strategy are still controversial. We report a patient who harbored a perforator aneurysm of basilar artery (PABA) presented as spontaneous subarachnoid hemorrhage (SAH) who recovered well after conservative observation.

## Case presentation

**Fig. 1:**
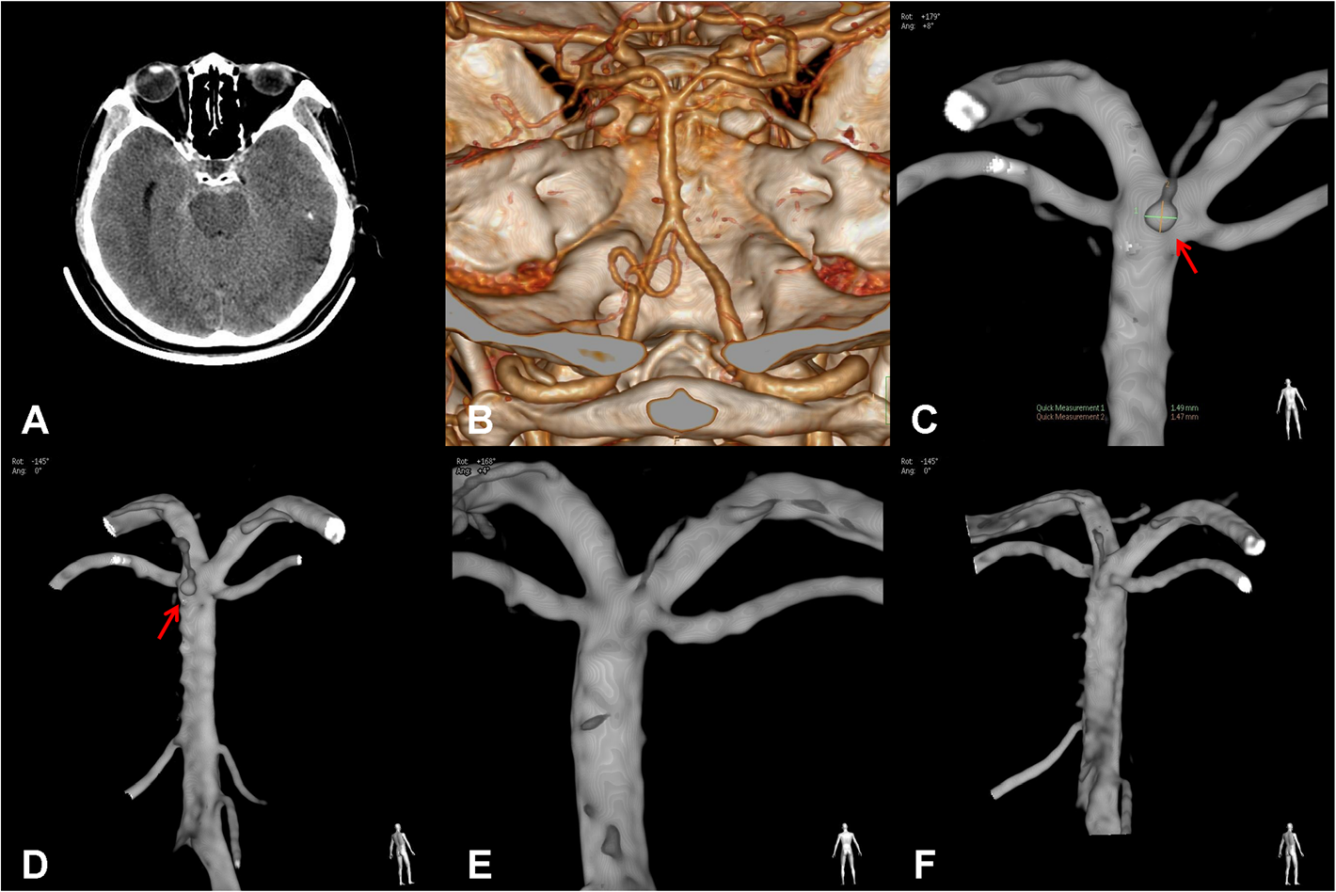
Imaging data of the case. **A** SAH surrounding potine was confirmed by head CT after onset. **B** Emergency CTA did not demonstrate definite source of hemorrhage. **C**, **D** Three-dimensional rotation angiography (3DRA) revealed a small aneurysm localized at posterior perforator of rostral basilar trunk. **E**, **F** The lesion disappeared from DSA during 2 months of follow-up with preservation of parent perforator

A 65-year-old gentleman had the history of hypertension and diabetes mellitus, which were well controlled for several years. In the emergency room, he complained of a sudden headache and dizziness for 5 days accompanied with nausea and vomiting. There were no neurological deficits detected by physical examination except for a positive meningeal irritation sign. Emergency computed tomography (CT) of the head showed SAH surrounding the pontine (Fig. 1A), but no aneurysm or vascular malformation was noted by CT angiography (CTA) (Fig. 1B). Digital subtraction angiography (DSA) revealed a small aneurysm less than 1.5 mm in diameter of a perforator which originated from the dorsal quandrification of the basilar trunk (Fig. 1C, D). Based on the CT images, this tiny lesion was considered a ruptured aneurysm. The patient experienced conservative observation and recovered well. A follow-up DSA 2 months later found that the aneurysm disappeared spontaneously and the parent perforator remained intact (Fig. 1E, F). The modified Rankin Scale (mRS) was 0 points for this patient at that time.

## Discussion

Van Gijn et al. reported a group of patients suffering from SAH in 1986 and presented the concept of “perimesencephalic nonaneurysmal subarachnoid hemorrhage (PNSH)” [2]. The similar clinical characteristics of these cases were that the location of SAH was mainly surrounding the pontine based on CT images and no definite vascular lesions could be detected by DSA. The rare pathogenic vein or capillary was considered the source of hemorrhage such as telangiectasia, tiny arteriovenous malformation, or arterial dissecting. Although the case we reported has the similar characteristic of CT images with that of PNSH, a small PABA is noted by DSA, which is definitely suggested that this is one of the causes of the so-called PNSH [3]. We retrieved the literatures from PubMed by the keywords as “basilar artery,” “perforator,” and “aneurysm.” As a result, 25 relevant literatures were selected and are listed in Table 1 after reviewing the abstracts. Due to the low morbidity of PABA, only 51 cases in 25 literatures have been published since the first case reported by Ghogawala et al. in 1996 [4]. PABA is mostly small and possibly thrombotic; therefore, it is difficult to make a definite diagnosis. For diagnosis, as the procedure of the case we reported, DSA is of choice and superior to CTA. Sometimes, shortly periodical follow-up by DSA is necessary [5].

**Table 1:** Results of document retrieval

First author (year)	n	Location^**a**^	Treatment	Method	Follow-up (month)	Prognosis
Ghogawala Z (1996) [4]	1	distal	microsurgery	clipping	6	good
Hamel W (2005) [5]	1	middle	microsurgery	EC & wrapping	7	mild ataxia
Fiorella D (2006) [6]	2	distal distal	endovascular treatment endovascular treatment	single stent stent-in-stent	unknown unknown	unknown unknown
Sanchez-Mejia RO (2007) [7]	3	distal middle middle	microsurgery microsurgery microsurgery	trapping & resection trapping & resection trapping & resection	unknown unknown unknown	good good good
Park SQ (2009) [3]	3	distal distal distal	conservative observation conservative observation conservative observation		16 16 1	good good good
Mathieson CS (2010) [8]	1	distal	microsurgery	clipping & resection	5	hydrocephalus & mild hypomnesia
Deshaies EM (2011) [9]	1	distal	endovascular treatment	stent-in-stent	6	good
Chen L (2012) [10]	2	middle middle	endovascular treatment endovascular treatment	coiling coiling	24 18	hemiplagia hemiplagia
Gross BA (2012) [11]	1	distal	microsurgery	clipping	12	hydrocephalus
Apok V (2013) [12]	1	distal	microsurgery	trapping & resection	unknown	hemiplagia & aphasia
Nyberg EM (2013) [13]	2	middle middle	endovascular treatment endovascular treatment	stent-in-stent stent-in-stent	14 4	good good
Ding D (2013) [14]	3	middle distal distal	conservative observation conservative observation endovascular treatment	Onyx occlusion	unknown 19 22	pontine infarction good hemiplagia
Sivakanthan S (2014) [15]	1	distal	microsurgery	clipping	6	good
Chalouhi N (2014) [16]	1	middle	endovascular treatment	flow diverter	6	good
Chavent A (2014) [17]	3	distal distal distal	conservative observation conservative observation conservative observation		6 12 6	good good good
Kim YJ (2014) [18]	1	distal	endovascular treatment	stent-in-stent	unknown	unknown
Daruwalla VJ (2016) [19]	1	distal	conservative observation		1.5	good
Peschillo S (2016) [20]	3	distal distal distal	endovascular treatment endovascular treatment endovascular treatment	flow diverter single stent & flow diverter flow diverter	6 36 6	mild monoplagia good mild hemiplagia
Forbrig R (2016) [21]	8	distal distal distal distal distal middle middle middle	conservative observation conservative observation endovascular treatment conservative observation endovascular treatment conservative observation conservative observation conservative observation	onyx occlusion coiling	6 6 60 5 23 11 15 78	hemiplagia & dysarthria good good hydrocephalus & mild disgnosia hydrocephalus & hemiplagia hydrocephalus & hemiplagia hydrocephalus & hemiplagia good
Satti SR (2016) [22]	1	distal	endovascular treatment	overlapping 3 stents	7	good
Aboukais R (2016) [23]	1	distal	conservative observation		1.5	good
Jiang Y (2016) [24]	1	proximal	endovascular treatment	microguidewire EC	6	good
Finitsis S (2017) [25]	4	distal distal middle middle	conservative observation conservative observation endovascular treatment conservative observation	flow diverter	1.5 12 3 1.5	good mild HP right dysacousis good
Buell TJ (2017) [26]	2	middle middle	endovascular treatment endovascular treatment	stent-in-stent stent-in-stent	unknown unknown	unknown unknown
Chau Y (2017) [27]	3	distal distal distal	conservative observation endovascular treatment endovascular treatment	stent-in-stent coiling stent-in-stent	12 6 6	good good good

^a^The basilar trunk was divided averagely into three parts – distal, middle and proximal segments

*n* number of cases, *EC* electrocoagulation

All cases harbored PABAs reported so far were presenting as SAH instead of incidental lesions, which resulted in its undefined natural history [27]. Difficulties of endovascular catheterization, microsurgical exposure, and preservation of parent perforator complicated the determination of therapeutic strategy. On the contrary, the disappearance of aneurysm from follow-up images was found in some cases without any endo- and extravascular procedures, which resulted that a periodical DSA follow-up was considered as a preferred strategy for many authors [17]. But in 21 cases who experienced conservative observation in the literatures, rehemorrhage occurred in 6 patients (28.57%). Two of them received microsurgery, and 2 of them were treated endovascularly. Of the above 4 cases, only 1 patient recovered well, and the rest 3 patients suffered from variant neurological deficits due to the ischemic events of parent perforators. Another 2 patients, who remained under observation, recovered well. Based on the above data, the prognosis of endovascular or microsurgical strategies may not be better than conservative observation [3, 7, 12, 21, 25]. And it appears to have comparable safety and efficacy outcomes to flow-diverter (FD) treatment of other ruptured basilar artery perforator aneurysms [28]. But according to previous reports, the conservative observation cases are at high risk of rebleeding [29]; for these cases, surgical treatment may be the best strategy. However, no matter traditional surgical clipping or endovascular intervention, the therapeutic effect is still controversial.

**Fig. 2:**
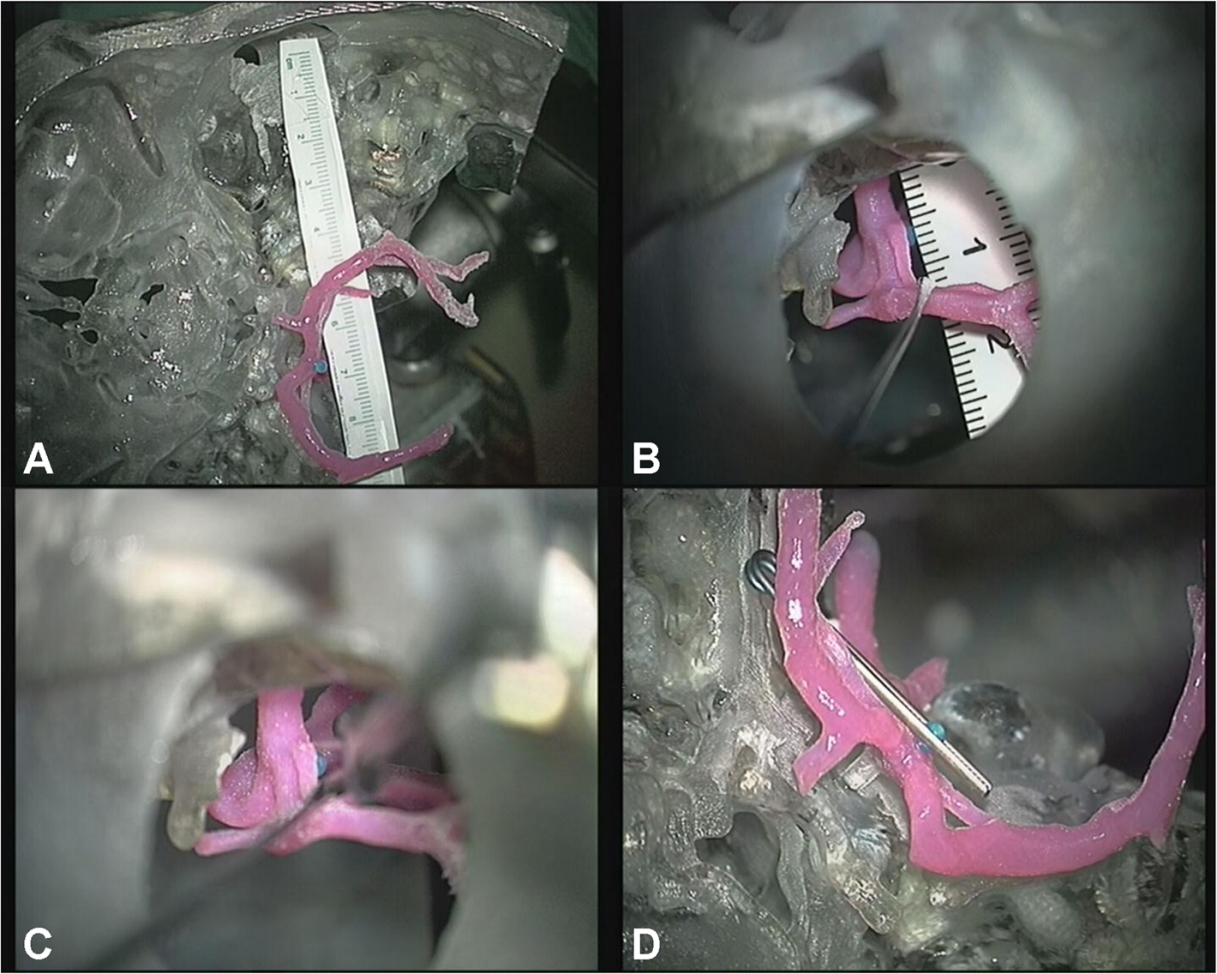
Simulating procedure on printed three dimension model. **A** Subtemporal keyhole approach of right side. The depth of aneurysm is 7 cm from temporal bone. **B** The length of visible basilar artery is 5 mm between petrosal apex and neck of aneurysm under the microsurgical corridor. **C** The aneurysm can be clipped by longer clip after retraction of isplateral superior cerebellar artery. **D** Relationship of clip and surrounding vascular structures after simulating clip application

Microsurgical clipping is not of choice for PABA. Sanchez-Mejia believed that such lesions usually had no definite neck and even were a type of blood blister-like aneurysm which should be trapped along with the parent perforator, and the control of the proximal basilar trunk was also difficult during microsurgery [7]. For our patient, we printed a three-dimensional model (Medprin Company, China, Guangzhou) to verify the feasibility of microsurgery [30]. During the simulating procedure, a more backward subtemporal approach was needed to expose the lesion, which increased the risk of injury of Labbé vein. Moreover, the aneurysm is located in the bottom of a narrow surgical corridor surrounded with critical nervous and vascular structures. In order to ensure that the clip will not obstruct the surgical view in this nearly 7 cm in-depth surgical approach, a longer straight clip is compulsory, which leads to the uncertainty of complete clipping of aneurysm and intact preserving of parent perforator (Fig. 2).

A tiny aneurysm is prone to rupture during superselective catheterization and the parent perforator is usually too small in caliber to be preserved. Of 5 cases who experienced endovascular embolization for such aneurysms by detachable coil (3 cases) or Onyx glue (Medtronic Company, USA, California) (2 cases) in the literature, only one case with distal BAPA recovered well, and the rest of the patients suffered from hemiplegia caused by ischemic events of perforator [10, 14, 21]. Although it has been reported that the parent perforators could be compensated [31], the safety of occlusion of these arteries is still undefined.

The hemodynamics of side-wall aneurysm model revealed that the blood flow velocity and wall shear stress in the aneurysmal cavity were significantly changed when the neck was covered by a stent. Furthermore, the denser the mesh, the greater the change [32]. Accordingly, flow diverter (FD) seems to be an ideal choice, but the incidence of infarction after stenting of posterior circulation aneurysm by FD is 14% [33]. Based on 5 relevant cases treated by FD (4 by PIPELINE [Medtronic Company, CA, USA] and 1 by SILK [Balt Extrusion Company, Montmorency, France]) reported in the literature, only one patient was free of ischemic events. It is suggested that excessive change of hemoynamics may be a risk factor of perforator occlusion [16, 20, 25]. Consequently, stent-in-stent technique with conventional stents (Enterprise [Codman Neuro Company, MA, USA], Neuroform [Stryker, MI, USA], Leo [Balt Extrusion Company, Montmorency, France], etc.) was considered as a better strategy to treat more PABAs. In 10 reported patients, half of them had no definite ischemic events; the prognosis of the rest of the patients was not reported as well [13, 22, 27]. However, whether the consequent antiplatelet therapy will increase the hemorrhagic complications remains to be solved by a study with larger sample size and longer follow-up.

Jiang et al. proposed a novel endovascular strategy [24]. They reported a case with a small perforator aneurysm of proximal basilar trunk which was failed to superselective catheterization. A microguidewire (Traxcess 14 [Microvention Terumo Company, CA, USA]) was positioned into the aneurysmal cavity followed by electrocoagulation with detaching box of Solitaire system (NDS-2, Medtronic, USA). As a result, the aneurysm was occluded completely; the parent perforator was preserved as well. This approach provides us with a new concept that seems to make the endovascular procedure for PABA more convenient, safe, and cost-effective. However, the long-term durability is still unknown. On the other hand, asymptomatic ischemic events caused by thrombosis during electrocoagulation should also be considered.

Ma et al. further reported three cases treated by endovascular electrothrombosis, which failed to pass an Echelon 10 microcatheter into the aneurysm [34]. A microguidewire (Traxcess-14 [MicroVention, Tustin, CA, USA]) was placed into the sac of the aneurysms through the microcatheter and connected its proximal tip to the Solitaire stent detachment system (ev3). The first case was conducted electrothrombosis at 4.0 V and 1.0 mA current three times for 30s each; the second one was conducted electrothrombosis at 4.0 V and 1.0 mA current three times (total of 3 min); the third one use the same parameter to sustained electrothrombosis for 1.5 min. As a result, in the first and the third cases, the aneurysms were successfully occluded without treatment-related complication. The second one failed and converted to endovascular coiling using a 1.3-F microcatheter. The patient suffered from brainstem infarction and finally died of severe SAH after surgery. At 6-month follow-up, the other two patients were neurologically intact and angiography showed total occlusion of both aneurysms. The findings in this report provide a potential treatment option for ruptured PABAs to prevent re-rupture, but the risk of perforator occlusion and aneurysm re-ruptured during surgery is still unknown, and the safety of this technique remains a concern. Therefore, it needs more research to confirm its safety and efficacy.

## Conclusions

Aneurysm that originated from a perforator of the basilar trunk was rare and difficult to treat. Conservative observation for certain cases with periodic angiography follow-up was considered in order to prevent the patients from potential iatrogenic effects.

## Data Availability

All data generated or analyzed during this study are included in this published article.

## Abbreviations

MCA: Middle cerebral artery
BAPA: Basilar artery perforator aneurysm
SAH: Subarachnoid hemorrhage
CT: Computed tomography
CTA: Computed tomography angiography
DSA: Digital subtraction angiography
mRS: Modified Rankin Scale
PNSH: Perimesencephalic nonaneurysmal subarachnoid hemorrhage
FD: Flow diverter
3DRA: Three-dimensional rotation angiography
